# Selective remodeling of glutamatergic transmission to striatal cholinergic interneurons after dopamine depletion

**DOI:** 10.1111/ejn.13715

**Published:** 2017-10-06

**Authors:** Jose de Jesus Aceves Buendia, Lior Tiroshi, Wei‐Hua Chiu, Joshua A. Goldberg

**Affiliations:** ^1^ Department of Medical Neurobiology Institute of Medical Research Israel – Canada The Faculty of Medicine The Hebrew University of Jerusalem 9112102 Jerusalem Israel

**Keywords:** 6‐OHDA, basal ganglia, optogenetics, slice electrophysiology

## Abstract

The widely held view that the pathophysiology of Parkinson's disease arises from an under‐activation of the direct pathway striatal spiny neurons (dSPNs) has gained support from a recently described weakening of the glutamatergic projection from the parafascicular nucleus (PfN) to dSPNs in experimental parkinsonism. However, the impact of the remodeling of the thalamostriatal projection cannot be fully appreciated without considering its impact on cholinergic interneurons (ChIs) that themselves preferentially activate indirect pathway spiny neurons (iSPNs). To study this thalamostriatal projection, we virally transfected with Cre‐dependent channelrhodopsin‐2 (ChR2) the PfN of Vglut2‐Cre mice that were dopamine‐depleted with 6‐hydroxydopamine (6‐OHDA). In parallel, we studied the corticostriatal projection to ChIs in 6‐OHDA‐treated transgenic mice expressing ChR2 under the Thy1 promoter. We found the 6‐OHDA lesions failed to affect short‐term synaptic plasticity or the size of unitary responses evoked optogenetically in either of these projections. However, we found that NMDA‐to‐AMPA ratios at PfN synapses—that were significantly larger than NMDA‐to‐AMPA ratios at cortical synapses—were reduced by 6‐OHDA treatment, thereby impairing synaptic integration at PfN synapses onto ChIs. Finally, we found that application of an agonist of the D_5_ dopamine receptors on ChIs potentiated NMDA currents without affecting AMPA currents or short‐term plasticity selectively at PfN synapses. We propose that dopamine depletion leads to an effective de‐potentiation of NMDA currents at PfN synapses onto ChIs which degrades synaptic integration. This selective remodeling of NMDA currents at PfN synapses may counter the selective weakening of PfN synapses onto dSPNs in parkinsonism.

## Introduction

The hypokinetic nature of Parkinson's disease (PD) is attributed, according to the prevailing clinical view, to a disruption of normal basal ganglia output. Loss of striatal dopamine results in a concurrent over‐activation of striatopallidal (indirect pathway) and under‐activation of striatonigral (direct pathway) spiny neurons (iSPNs and dSPNs, respectively; Albin *et al*., 1995; Wichmann & DeLong, 1996). This clinical view has gained firm experimental grounding in studies that demonstrate that dopamine depletion leads to a) the bidirectional modulation of SPN firing rates (Mallet *et al*., 2006); b) a selective remodeling of intrinsic and synaptic properties of iSPNs (but not dSPNs)(Day *et al*., 2006)—probably reflecting a homeostatic response to iSPN over‐activation; and that c) selective optogenetic activation of dSPNs can alleviate motor symptoms in experimental parkinsonism (Kravitz *et al*., 2010). The basal activation of SPNs arises extrinsically from convergent glutamatergic inputs from the cortex and the parafascicular nucleus (PfN) of the thalamus (Wilson *et al*., 1990; Lapper & Bolam, 1992; Thomas *et al*., 2000; Reynolds & Wickens, 2004; Plotkin *et al*., 2011) raising the possibility that these inputs are remodeled in PD, as well. Indeed, recent studies of the synaptic properties of cortico‐ and thalamostriatal synapses onto SPNs in dopamine‐depleted mice have found selective amplification of NMDA currents in corticostriatal synapses onto iSPNs (Warre *et al*., 2011) or a selective weakening of thalamostriatal synapses onto dSPNs (Parker *et al*., 2016) that could contribute to the imbalance between these pathways in PD.

However, to fully appreciate the impact of these two glutamatergic projections on SPN pathophysiology, it is necessary to consider their impact on striatal interneurons (Sciamanna *et al*., 2015; Assous *et al*., 2017) including cholinergic interneurons (ChIs) which they innervate (Wilson *et al*., 1990; Lapper & Bolam, 1992; Bennett & Wilson, 1998; Thomas *et al*., 2000; Matsumoto *et al*., 2001; Zackheim & Abercrombie, 2005; Doig *et al*., 2014) and that exert powerful and multifaceted control over striatal circuitry (Koós & Tepper, 2002; Goldberg *et al*., 2012; Silberberg & Bolam, 2015; Assous *et al*., 2017). First, ChIs influence SPN dynamics via strong di‐synaptic inhibition (English *et al*., 2011; Nelson *et al*., 2014; Faust *et al*., 2016). Second, PfN and cortical inputs can activate ChIs synchronously and thereby induce striatal dopamine release by activating nicotinic acetylcholine receptors (nAChRs) on dopamine fibers (Threlfell *et al*., 2012; Kosillo *et al*., 2016). Third, by activating muscarinic acetylcholine receptors (mAChRs), ChIs modulate SPN excitability, synaptic transmission and synaptic plasticity (Pisani *et al*., 2007; Goldberg *et al*., 2012; Hernandez‐Flores *et al*., 2014). Of particular relevance is the fact that the PfN projection to ChIs gates corticostriatal input (Pakhotin & Bracci, 2007) to bias striatal dynamics toward preferential activation of iSPNs (Ding *et al*., 2010; Mamaligas & Ford, 2016). Thus, a functional re‐wiring of thalamostriatal projection to SPNs in PD (Parker *et al*., 2016) that alters the balance between dSPNs and iSPNs cannot be fully appreciated without considering any putative remodeling of the glutamatergic transmission to ChIs under the same conditions. Such a remodeling of thalamostriatal input to ChIs may contribute to their loss of responsiveness to salient input (Matsumoto *et al*., 2001) and could underlie PD patients’ difficulty in attending to salient stimuli.

In this study, we used optogenetics to selectively activate the PfN projection to the striatum and studied synaptic transmission in acute striatal slices from control and dopamine‐depleted mice. For comparison, we studied the effect of the dopamine depletion on corticostriatal transmission to ChIs using a transgenic mouse that expresses channelrhodopsin‐2 (ChR2) in nominally cortical (non‐thalamic) fibers that we used previously to describe remodeling of this projection in a model of Huntington's disease (HD) (Tanimura *et al*., 2016).

## Materials and methods

### Animals

This study was carried out in accordance with the recommendations of and approved by the Hebrew University Animal Care and Use Committee. All of the experiments were conducted with 2–4‐month‐old male mice. To investigate thalamostriatal transmission to ChIs, we used Vglut2‐ires‐Cre mice (stock number 016963; Jackson Laboratories, Bar Harbor, ME, USA), and to investigate corticostriatal transmission to ChIs, we used homozygous transgenic Thy1‐ChR2 mice [B6.Cg‐Tg (Thy1‐COP4/EYFP) 18Gfng/1] expressing ChR2 under the Thy1 promoter (Arenkiel *et al*., 2007).

### Surgical procedures

Mice were deeply anesthetized with isoflurane in a non‐rebreathing system (2.5% induction, 1–1.5% maintenance) and placed in a stereotaxic frame (Kopf Instruments, Tujunga, CA, USA). Temperature was maintained at 35 °C with a heating pad, artificial tears were applied to prevent corneal drying, and animals were hydrated with a bolus of injectable saline (10 mL/kg) mixed with analgesic (5 mg/kg carprofen). Bilateral stereotaxic injections into caudal intralaminar nuclei of thalamus were performed under aseptic conditions. Adeno‐associated viruses (AAVs) serotype 9 carrying double‐floxed fusion genes for hChR2 (E123A) and eYFP under an EF1a promoter (University of Pennsylvania Vector Core, Addgene #35507) were used to transfect PfN neurons. Injection coordinates that target PfN were adapted from Ellender and co‐workers (Ellender *et al*., 2013) and were from Bregma: lateral, 0.65 mm; posterior, 2.33 mm; and 3.45 mm depth from surface of brain (Paxinos & Franklin, 2004). A small hole was bored into the skull with a micro‐drill bit, and a glass pipette was slowly inserted at the PfN coordinates. To minimize backflow, solution was slowly injected; a total volume of 280 nL (>2.5 × 10^12^ GC/mL) of the AAV constructs was injected over a period of approximately 1.5 min, and the pipette was left in place for 5 min before slowly retracting it.

To deplete dopamine, we unilaterally injected during the same surgery 3 μg 6‐hydroxydopamine (6‐OHDA, Sigma‐Aldrich) dissolved in 360 nl of injectable 0.9% saline/0.2% ascorbic acid solution into the medial forebrain bundle. Injection coordinates were from Bregma: lateral, 1.1 mm; posterior, 0.7 mm; and 4.8 mm depth from surface of brain. Desipramine (25 mg/kg) was injected *i.p*. prior to the surgery to protect noradrenergic neurons. For a few days following surgery until the weight of the mice stabilized, we injected lactated Ringer's (0.1 mL/g) *i.p*. and added sucrose to the drinking water (30%) to help the mice recover. Non‐lesioned mice served as controls.

### Slice preparation

Two to three weeks after the viral injections, mice were deeply anesthetized with ketamine (200 mg/kg)–xylazine (23.32 mg/kg) and perfused transcardially with ice‐cold‐modified artificial cerebrospinal fluid (ACSF) bubbled with 95% O_2_–5% CO_2_, and containing (in mm) 2.5 KCl, 26 NaHCO_3_, 1.25 Na_2_HPO_4_, 0.5 CaCl_2_, 10 MgSO_4_, 0.4 ascorbic acid, 10 glucose and 210 sucrose. The brain was removed, and sagittal slices sectioned at a thickness of 240 μm were obtained in ice‐cold‐modified ACSF. Slices were then submerged in ACSF, bubbled with 95% O_2_–5% CO_2_, and containing (in mm) 2.5 KCl, 126 NaCl, 26 NaHCO_3_, 1.25 Na_2_HPO_4_, 2 CaCl_2_, 2 MgSO_4_ and 10 glucose, and stored at room temperature for at least 1 h prior to recording.

### Slice visualization, electrophysiology and optogenetic stimulation

The slices were transferred to the recording chamber mounted on an Olympus BX51 upright, fixed‐stage microscope and perfused with oxygenated ACSF at room temperature. A 60X, 0.9 NA water immersion objective was used to examine the slice using Dodt contrast video microscopy. Patch pipette resistance was typically 3–4 MΩ when filled with recording solutions. In voltage clamp experiments, the intracellular solution contained (in mm) 127.5 CsCH_3_SO_3_, 7.5 CsCl, 10 HEPES, 10 TEA‐Cl, 4 phosphocreatine disodium, 0.2 EGTA, 0.21 Na_2_GTP and 2 Mg_1.5_ATP (pH = 7.3 with CsOH, 280–290 mOsm/kg). For whole‐cell current clamp recordings, the pipette contained (in mm) 135.5 KCH_3_SO_4_, 5 KCl, 2.5 NaCl, 5 Na‐phosphocreatine, 10 HEPES, 0.2 EGTA, 0.21 Na_2_GTP and 2 Mg_1.5_ATP (pH = 7.3 with KOH, 280–290 mOsm/kg).

Electrophysiological recordings were obtained with a MultiClamp 700B amplifier (Molecular Devices, Sunnyvale, CA). Junction potential, which was 7–8 mV, was not corrected. Signals were digitized at 10 kHz and logged onto a personal computer with the Winfluor software (John Dempster, University of Strathclyde, UK). Blue‐light LED (470 nm; Mightex, Toronto, ON, Canada) was used for full‐field illumination via the objective. Single pulses were 1 ms long. Optogenetic paired pulses were 100 ms apart. Pulse trains were 5 pulses long at 10 Hz or 12 pulses long at 25 Hz. We chose an LED intensity that generated excitatory postsynaptic potentials (EPSPs) with amplitudes in the 1–5 mV range and then kept that intensity constant for all the experiments described herein.

### Histology

After the electrophysiological recordings, slices were fixed by immersion overnight in 4% paraformaldehyde followed by 30% sucrose. The sections were re‐sectioned by inclusion in agar from 240 to 50 μm. The thin slices were washed three times for 5 min in 0.1 m phosphate buffer and incubated in anti‐rabbit tyrosine hydroxylase (TH) primary antibody, 1:1,000 (Millipore, Billerica, MA, USA) overnight. On the next day, the slices were washed again, incubated for 2 h in a Alexa fluor 647 conjugated secondary antibody, 1:1,000 (Abcam, Cambridge, UK), plated and cover‐slipped for confocal imaging on a Zeiss LSM 510 Meta system.

Because the dopamine lesions were so extensive, the striatal TH immunofluorescence signal was typically many of order of magnitudes weaker in slices from the lesioned mice relative to the control ones. Thus, to quantify the difference in the extent of immunofluorescence between sagittal images of control and lesioned striata, we set the laser intensity so that the background signal (e.g., in the corpus callosum that should nominally exhibit no TH immunoreactivity) in both control and lesioned would be within—but at the lower end of—the dynamic range of the confocal microscope. This inevitably caused the immunofluorescent signal in the control striata to saturate, precluding the quantification of the *level* of TH immunoreactivity in control relative to lesioned striate. Instead, we quantified the *extent* of the lesion using the following statistic. For each TH‐stained sagittal image, we first determined the mean pixel intensity in a square region (112 μm each side) within the corpus callosum. We then chose a square region within the striatum (558 μm each side) that contained 202, 500 pixels. The statistic is defined as the percentage of the pixels that had intensity values that were larger than 3 times the mean pixel intensity measured in the corpus callosum.

### Drugs and reagents

All experiments were conducted with a cocktail of synaptic blockers for acetylcholine and GABA receptors including (in μm) 10 atropine, 10 mecamylamine, 2 CGP 55845, 10 SR 95531. In some experiments, we used D‐APV (50 μm), an NMDA receptors (NMDAR) antagonist, and R(+)‐SKF‐81297 (1 μm), a D_1_‐like receptor agonist. All drugs and reagents were acquired from Tocris (Ellisvile, MO, USA) or Sigma (St. Louis, MO, USA).

### Data analysis and statistics

Data were analyzed, and curve fitting was performed using custom‐made code in MATLAB (MathWorks, Natick, MA, USA). To calculate optogenetic paired‐pulse ratios (PPRs), average current waveforms in response to paired pulses were acquired. The PPR was defined as the ratio of the peak of the 2nd averaged excitatory postsynaptic current (EPSC) was divided by the peak of the 1st averaged EPSC. By tuning the 470‐nm LED intensity so that failures and successes occur stochastically, we were able to estimate the amplitudes of the unitary EPSCs, by fitting a mixture of Gaussian model (Redman, 1990; Bekkers & Clements, 1999) to the empirical probability distribution function of the EPSC amplitudes (Tanimura *et al*., 2016). NMDA‐to‐AMPA (NMDA:AMPA) ratios were defined as the ratio of the peak averaged outward current, when the Cs^+^‐loaded ChIs were held at +40 mV, divided by the peak averaged outward current at the time of the peak of the AMPA current, both elicited by brief 470‐nm pulses. The AMPA peak was either self‐evident in the outward current waveform and/or was revealed after antagonizing the NMDA current with D‐APV. To compare the degree of synaptic integration among ChIs, they were hyperpolarized to quiescence in current clamp mode and their averaged excitatory postsynaptic potentials (EPSPs) in response to either 5 pulses at 10 Hz or 12 pulses at 25 Hz (Kosillo *et al*., 2016) were normalized to the amplitude of the 1st EPSP in the train.

The nonparametric two‐tailed Wilcoxon rank‐sum test (RST) was used for independent samples, and the nonparametric two‐tailed Wilcoxon signed‐rank test (SRT) was used for matched samples. Boxplots represent range (whiskers), median (thick bar) and lower and upper quartiles. The parametric ancova test was used to test significant changes in these curves. Null hypotheses were rejected if the *P*‐value was below 0.05.

## Results

### Short‐term plasticity at PfN and cortical synapses onto ChIs were unaltered by 6‐OHDA lesions

**Figure 1:**
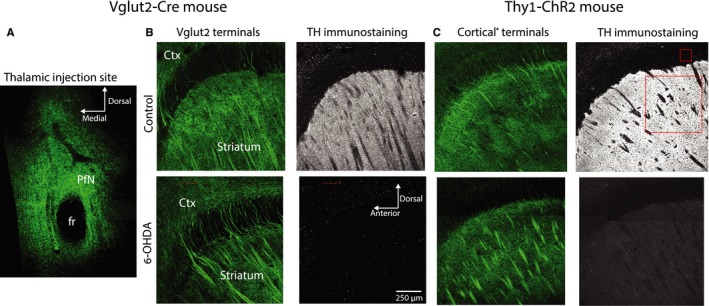
Distribution of fibers arising from the parafascicular nucleus (PfN) or the cortex in control and dopamine‐depleted striata. (A) Confocal image of a coronal slice through the site of AAV injections in the PfN of Vglut2‐Cre mice. The green signal arises from the eYFP conjugated to the ChR2 harbored in the AAV. The dark spot is the fasciculus retroflexus (fr). (B) Left: Expression of eYFP in Vglut2 fibers arising from the PfN in sagittal slices of the striatum of control (top) and 6‐OHDA‐lesioned (bottom) Vglut2‐Cre mice. Ctx—cortex. Right: Tyrosine hydroxylase (TH) immunoreactivity in control (top) and 6‐OHDA‐lesioned (bottom) Vglut2‐Cre mice. Left: Expression of eYFP in nominally cortical (hence the asterisk) fibers in sagittal slices of the striatum of control (top) and 6‐OHDA‐lesioned (bottom) Thy1‐ChR2 mice. C. Right: TH immunoreactivity in control (top) and 6‐OHDA‐lesioned (bottom) Thy1‐ChR2 mice. Small and large red boxes represent the areas within the corpus callosum and the striatum, respectively, used to assess the extent of the reduction in TH immunoreactivity after dopamine depletion.

To study synaptic transmission from the PfN to striatal ChIs, we injected AAVs that harbor Cre‐dependent ChR2 into the PfN of adult C57BL/6J mice that express Cre‐recombinase under the Vglut2 promoter (Fig. 1A; Fremeau *et al*., 2004; Smith *et al*., 2014; Parker *et al*., 2016) resulting in widespread ChR2 expression in the dorsal striatum 2‐3 weeks following transfection (Fig. 1B). Measurements of the excitatory synaptic currents (EPSCs) were conducted at this time point in acute slices of dorsal striatum. During the viral injection surgery, some mice underwent an additional 6‐OHDA injection into the medial forebrain bundle to render them hemi‐parkinsonian. ChIs receive glutamatergic synapses from cortex as well (Wilson *et al*., 1990; Bennett & Wilson, 1998). Thus, to test for pathway specific adaptations in glutamatergic inputs, we conducted parallel experiments in transgenic mice that express ChR2 in cortical fibers (Fig. 1C) (Tanimura *et al*., 2016). Only cells from slices that were verified *post hoc* to exhibit near complete loss of striatal TH positive fibers were retained for the analysis. Quantification of the extent of the lesion (see Materials and Methods: Histology) demonstrated that in intact striata (*n *= 8 mice), a median of 96% of pixels (Fig. 1C, right, large red box) exhibited TH immunoreactivity that was at least 3 times brighter than the level exhibited in the corpus callosum (that should nominally exhibit no TH immunoreactivity, Fig. 1C, right, small red box). In contrast, in the lesioned striata (*n *= 7 mice), this percentage dropped to a median of 0.015% of the pixels (*P *= 2/6435 ≈ 0.0003, RST).

**Figure 2:**
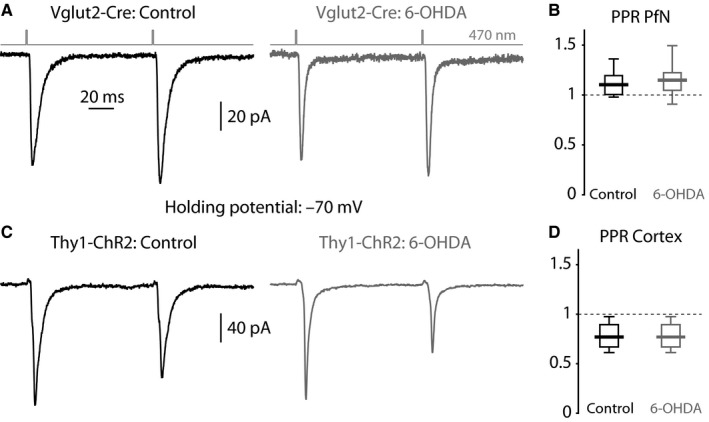
Optogenetic PPRs in ChIs from control and dopamine‐depleted striata. (A) Averaged EPSCs evoked in ChIs by a pair of 470‐nm light pulses (100‐ms inter‐pulse interval) that activate ChR2‐laden PfN fibers from a control (left) and 6‐OHDA‐lesioned (right) Vglut2‐Cre mouse. (B) Boxplot of PPRs at PfN synapses onto ChIs in control and 6‐OHDA‐lesioned mice. (C) Averaged EPSCs evoked in ChIs by a pair of 470‐nm light pulses that activate ChR2‐laden fibers from a control (left) and 6‐OHDA‐lesioned (right) Thy1‐ChR2 mouse. (D) Boxplot of PPRs at nominally cortical synapses onto ChIs in control and 6‐OHDA‐lesioned mice.

Optogenetic activation of Vglut2‐expressing fibers (arising from the PfN) generated EPSCs in Cs^+^‐loaded ChIs. To test for a possible effect of dopamine depletion on short‐term plasticity, we measured the effect of the 6‐OHDA lesion on the optogenetic paired‐pulse ratios (PPRs) (Fig. 2A). As reported previously, PPRs in control mice were larger than unity (median control: 1.10, *n *= 13 neurons, *N *= 9 mice) indicating that PfN synapses are facilitating (Tanimura *et al*., 2016). The 6‐OHDA lesion did not alter the PfN PPRs (Fig. 2B; median 6‐OHDA: 1.15, *n *= 10 neurons, *N *= 6 mice; *P *= 0.73, RST). Optogenetic PPRs in corticostriatal synapses (Fig. 2C) were less than unity (Fig. 2D; median control: 0.84, *n *= 7 neurons, *N *= 4 mice), as shown previously (Tanimura *et al*., 2016), indicating a depressing synapse, and were not affected by 6‐OHDA, either (Fig. 2B; median 6‐OHDA: 0.77, *n *= 7 neurons, *N *= 2 mice; *P *= 0.46, RST). Thus, in acute striatal slices in which dopamine tone is presumably markedly reduced, we found no evidence that dopamine depletion alters presynaptic probability of release at either glutamatergic synapse onto ChIs.

### Unitary responses at PfN and cortical synapses onto ChIs were unaltered by 6‐OHDA lesions

**Figure 3:**
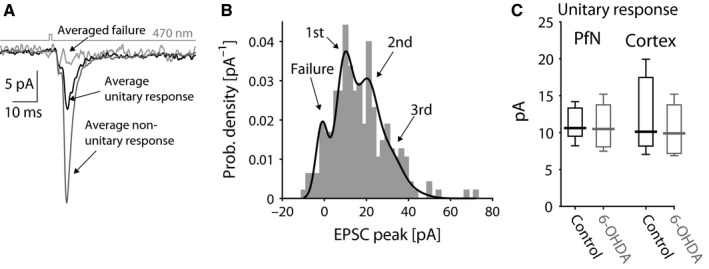
Minimal optogenetic stimulation of glutamatergic inputs to ChIs from control and dopamine‐lesioned striata. (A) Averaged unitary (black) and non‐unitary (dark gray) responses using a minimal stimulation protocol where the presence or absence (light gray) of a response is stochastic. (B) Empirical probability density function (pdf) of the amplitude of the stochastically generated EPSCs (corresponding to the experiment depicted in panel A) is fit by a binomial mixture of Gaussian model. (C) Boxplot of unitary response extracted from the value of the first mode of the pdf fit to each experiment demonstrates a lack of difference between PfN and cortical synapses and a lack of change following dopamine depletion.

To estimate the postsynaptic efficacy of transmission at individual synapses, we used a minimal optogenetic stimulation protocol (Boyd *et al*., 2012), which is a measure of AMPAR conductance, or number of receptors per synapse (Béïque & Andrade, 2003). This experiment (see Methods) demonstrated that the amplitude of the unitary responses at PfN synapses (median PfN unitary EPSC: 11.5 pA, *n *= 8 neurons, *N *= 6 mice) was no different from the amplitude of unitary responses at cortical synapses (median cortical unitary EPSC: 10.5 pA, *n *= 5 neurons, *N *= 2 mice; Tanimura *et al*., 2016). Moreover, these amplitudes were unaffected by the 6‐OHDA lesion (Fig 3; PfN: 10.6 pA, *n *= 5 neurons, *N *= 2 mice, *P *= 0.83, RST; cortex: 10.3 pA, *n *= 4 neurons, *N *= 3 mice**,** *P *= 0.56, RST). These findings indicate that the reported difference in synaptic strength between PfN and cortical neurons onto ChIs (Lapper & Bolam, 1992; Ding *et al*., 2010) is not attributable to a difference in the number of AMPARs at these respective synapses and suggests that dopamine depletion does not affect AMPAR trafficking (Tritsch & Sabatini, 2012).

### 6‐OHDA lesions reduce NMDA‐to‐AMPA ratios at PfN synapses only

**Figure 4:**
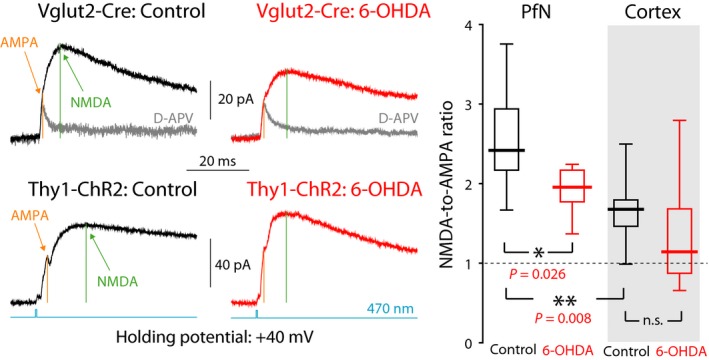
NMDA currents are relatively larger for PfN than for cortical input to ChIs and are reduced at PfN synapses onto ChIs following dopamine depletion. Left: Averaged optogenetic EPSC evoked in ChIs clamped at +40 mV due to PfN input in a control Vglut2‐Cre mouse (top, left), and in a 6‐OHDA‐lesioned Vglut2‐Cre mouse (top, right); nominally cortical input in a control Thy1‐ChR2 mouse (bottom, left), and in a 6‐OHDA‐lesioned Thy1‐ChR2 mouse (bottom, right). Light gray traces are the responses after application of D‐APV, an NMDAR antagonist. Right: Boxplots of NMDA‐to‐AMPA ratios at cortical and PfN synapses in control mice, and 6‐OHDA‐lesioned mice, reveal that the NMDA component at PfN synapses is significantly larger than at cortical synapses onto ChIs and is reduced following 6‐OHDA lesions. n.s.—not significant.

Previous work has shown that intrastriatal acetylcholine (Consolo *et al*., 1996a) and dopamine (Kosillo *et al*., 2016) release evoked by activation of PfN fibers is more strongly dependent on activation of NMDA receptors (NMDARs) on ChIs than when evoked by activation of cortical fibers. Accordingly, synaptic integration by ChIs of PfN input was shown to be more dependent on NMDA currents than synaptic integration of cortical inputs (Kosillo *et al*., 2016). These findings strongly suggest that PfN synapses onto ChIs have a larger NMDA component. Indeed, direct measurement of the NMDA‐to‐AMPA ratio (NMDA:AMPA) revealed that it was significantly larger at PfN synapses (PfN median control NMDA:AMPA: 2.42, *n *= 9 neurons, *N *= 7 mice) than at cortical synapses (cortex median control NMDA:AMPA: 1.68 *n *= 11 neuron, *N *= 7 mice; *P *= 0.008, RST) onto ChIs (Fig 4).

D_1_ dopamine receptors are known to potentiate NMDA currents in the striatum (Levine *et al*., 1996; Cepeda *et al*., 1998; Flores‐Hernandez, 2002; Hallett *et al*., 2005; Jocoy, 2011; Warre *et al*., 2011; Tritsch & Sabatini, 2012; Cahill *et al*., 2014). Additionally, whole‐cell NMDA currents elicited in ChIs by intrastriatal stimulation (which likely activated both cortical and PfN synapses) were reduced in 6‐OHDA‐lesioned mice (Feng *et al*., 2014) and MPTP‐lesioned primates (Hallett *et al*., 2005). Therefore, it is possible that there would be a differential effect of 6‐OHDA lesions on NMDA vs. AMPA currents, leading to a reduction in the NMDA‐to‐AMPA ratio at PfN synapses. Measurement of the NMDA‐to‐AMPA ratios at PfN synapses onto ChIs in 6‐OHDA mice revealed (Fig. 4) a significant reduction relative to controls (PfN median 6‐OHDA NMDA:AMPA: 1.96, *n *= 6 neurons, *N *= 3 mice; *P *= 128/5005 ≈ 0.026, RST). In contrast, the ratio at cortical synapses was unchanged by 6‐OHDA treatment (cortex median 6‐OHDA NMDA:AMPA: 1.14, *n *= 7 neurons, *N *= 4 mice; *P *= 0.13, RST).

### 6‐OHDA lesions impair synaptic integration of PfN inputs in ChIs

**Figure 5:**
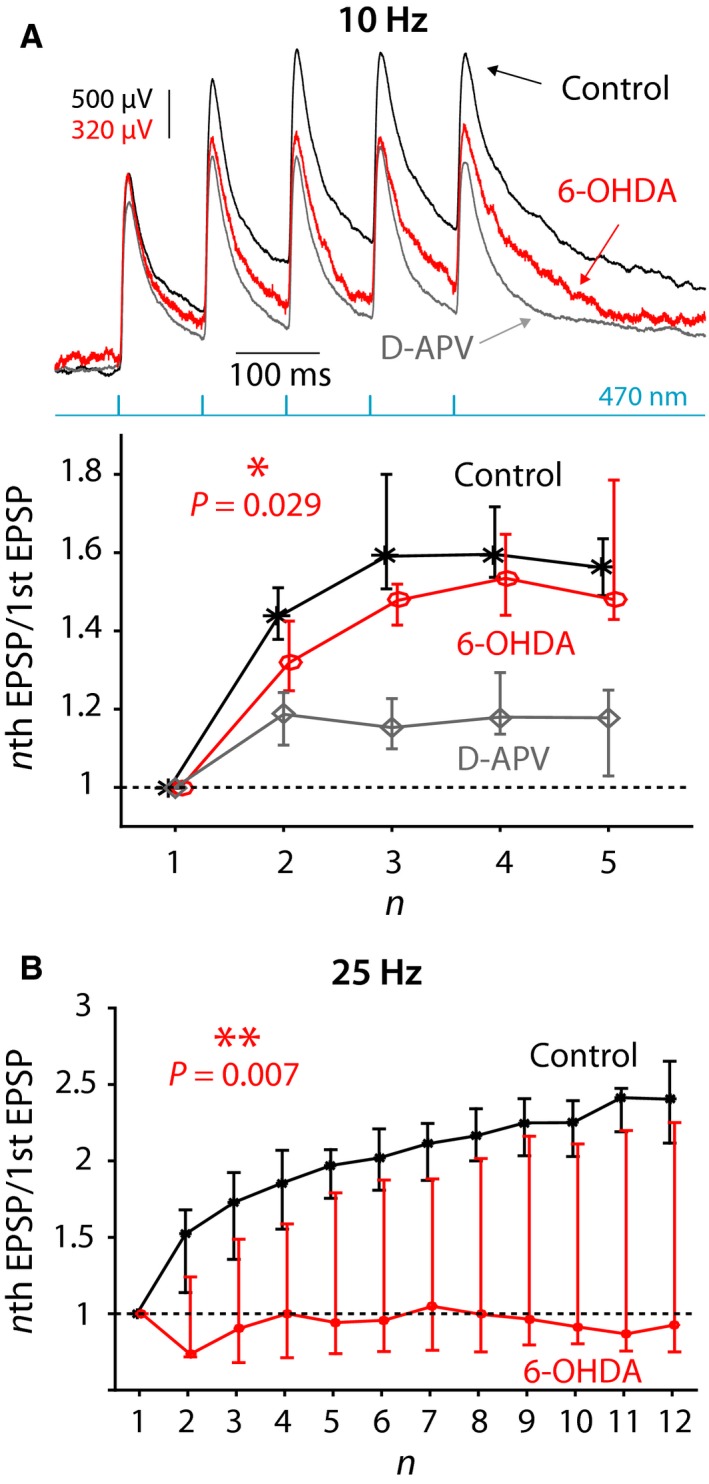
Synaptic integration at PfN synapses onto ChIs is degraded following 6‐OHDA treatment. (A) Top: Synaptic integration at PfN synapses onto ChIs in response to five 470‐nm pulses at 10 Hz in a control mouse before (black) and after (gray) application of D‐APV and in 6‐OHDA‐lesioned mouse (red). Note the difference in scale between the red vs. the other traces chosen so as to align the amplitude of the first EPSP. Bottom: Ratio of the amplitude of n^th^ EPSP to the 1st EPSP in control mice before (black) and after (gray) application of D‐APV and in 6‐OHDA‐lesioned mice (red). (B) Same as in panel B, bottom, except that 12 pulses are given at 25 Hz. The curves in panels B and C depict medians and confidence intervals given by the *50%×* (*1 ± 1/√*k) quantiles, where k is the sample size (Lasser‐Katz *et al*., 2017).

The variability in the amplitude of the EPSCs, resulting from the variable degree of viral transfection among the striatal slices, precluded reliably measuring changes in the amplitudes of either NMDA or AMPA EPSCs individually following dopamine depletion, and we indeed failed to observe any such changes in our data (not shown). Therefore, the observed reduction in the NMDA‐to‐AMPA ratio at PfN synapses onto ChIs following 6‐OHDA treatment could, in principle, mean that either AMPA currents are upregulated and/or NMDA currents are downregulated. However, in light of the previous report of a reduction in whole‐cell NMDA currents in ChIs from 6‐OHDA‐lesioned mice (Feng *et al*., 2014), and in light of the fact that the manipulation of dopamine receptors generally does not affect AMPAR trafficking in the striatum (Tritsch & Sabatini, 2012), it seems likely that 6‐OHDA treatment reduced the NMDA current. Furthermore, a reduction in the NMDA current—but not an increase in the AMPA current—should impair temporal summation of PfN inputs. To test this, we recorded K^+^‐loaded ChIs in whole‐cell current clamp, hyperpolarized them so that they cease to fire and measured the degree of synaptic integration in control and 6‐OHDA‐lesioned mice. When stimulating at 10 Hz, there was a minor (but statistically significant) reduction in efficacy of synaptic integration (*P *= 0.029, ancova, control: *n *= 9 neurons, *N *= 6 mice; 6‐OHDA: *n *= 14 neurons, *N *= 6 mice) that approaches the degree of integration when NMDARs are blocked with D‐APV (Fig. 5A). The degradation of synaptic integration in the 6‐OHDA‐lesioned mice became much more evident when stimulating at 25 Hz (Fig. 5B, *P *= 0.007, ancova, control: *n *= 6 neurons, *N *= 4 mice; 6‐OHDA: *n *= 7 neurons, *N *= 3 mice). We conclude that the reduction in NMDA‐to‐AMPA ratios leads to an impairment of synaptic integration of PfN input to ChIs in 6‐OHDA‐lesioned mice.

### D_5_ receptors potentiate NMDA currents selectively at PfN synapses on ChIs

**Figure 6:**
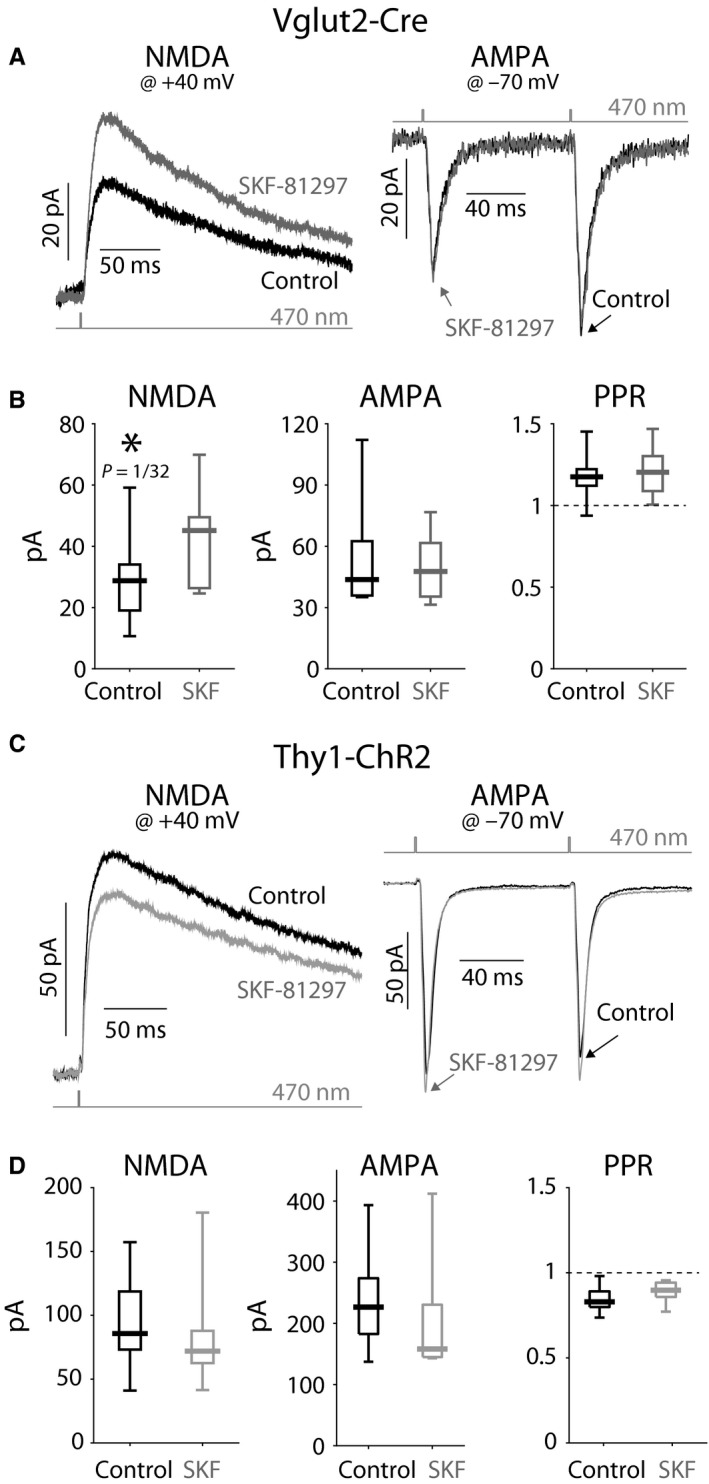
NMDAR currents at PfN—but not cortical—synapses onto ChIs are potentiated by D_5_R activation. (A) Averaged optogenetic EPSC from PfN fibers in Vglut2‐Cre mice evoked in ChIs clamped at +40 mV (left) and ‐70 mV (right) before (black) and after (gray) application of SKF‐81297, a D_1_‐like receptor agonist. (B) Boxplots of the amplitude of the NMDA component (left, measured at +40 mV); the AMPA component (middle, 1st EPSC, measured at ‐70 mV); and the PPRs (right) demonstrate a significant amplification of the NMDA current without a concurrent change in the AMPA current or a change in the PPRs. (C) Averaged optogenetic EPSC from nominally cortical fibers in Thy1‐ChR2 mice evoked in ChIs clamped at +40 mV (left) and ‐70 mV (right) before (black) and after (gray) application of SKF‐81297. B. Boxplots of the amplitude of the NMDA component (left, measured at +40 mV); the AMPA component (middle, 1st EPSC, measured at −70 mV); and the PPRs (right) failed to demonstrate any change in the NMDA or AMPA currents or a change in the PPRs.

Why would dopamine depletion lead to downregulation of NMDA currents? NMDARs are potentiated in SPNs by activation of D_1_‐like receptors (Flores‐Hernandez, 2002; Jocoy, 2011; Tritsch & Sabatini, 2012). ChIs express D_5_ (D_1_‐like) receptors (D_5_Rs) (Bergson *et al*., 1995; Yan & Surmeier, 1997), and it is, therefore, possible that NMDA currents are potentiated in ChIs via activation of these receptors, as well (Consolo *et al*., 1996b). To test this hypothesis, we measured the effect of 1 μm SKF‐81297, a D_1_‐like (*i.e.,* D_1_/D_5_) receptor agonist, on the amplitude of the optogenetically evoked NMDA and AMPA currents evoked in control mice (Fig. 6A). We found that SKF‐81297 significantly potentiated the NMDA currents recorded at +40 mV (median control: 28.8 pA; median SKF‐81297: 45.1 pA, *n *= 7 neurons, *N *= 4 mice; *P *= 1/32, SRT). Importantly, application of the D_1_‐like receptor agonist failed to change the amplitude of the AMPA EPSCs (median control: 43.7 pA; median SKF‐81297: 47.7 pA, *n *= 7 neurons, *N *= 4 mice; *P *= 0.58, SRT) or the PPRs (median control: 1.18; median SKF‐81297: 1.20, *n *= 7 neurons, *N *= 4 mice; *P *= 3/8, SRT) recorded at −70 mV, indicating that the potentiation is postsynaptic and selective to NMDA currents (Fig. 6B).

Interestingly, the potentiation of NMDARs by the D_1_‐like receptor agonist was selective to PfN synapses onto ChIs. Measurement of NMDA and AMPA currents at cortical synapses (Fig. 6C) onto ChIs demonstrated that they were unchanged by SKF‐81297 (median control: 85.7 pA; median SKF‐81297: 79.1 pA, *n *= 7 neurons, *N *= 2 mice; *P *= 0.30, SRT). Here too, application of the D_1_‐like receptor agonist did not affect the amplitude of the AMPA EPSCs (median control: 227 pA; median SKF‐81297: 158 pA, *n *= 7 neurons, *N *= 2 mice; *P *= 7/32 ≈ 0.22, SRT) or the PPRs (median control: 0.83; median SKF‐81297: 0.90, *n *= 7 neurons, *N *= 2 mice; *P *= 5/64 ≈ 0.078, SRT) recorded at −70 mV (Fig. 6D). Taken together, these results suggest that the reduction in the NMDA‐to‐AMPA ratio following dopamine depletion may be attributable to the loss of tonic activation of D_5_Rs, which effectively de‐potentiates NMDARs—selectively at PfN synapses onto ChIs—thereby impairing synaptic integration at these synapses.

## Discussion

The central finding of our study is that depletion of striatal dopamine leads to a reduction in the NMDA‐to‐AMPA ratio at PfN synapses onto ChIs. We also demonstrated that the NMDA‐to‐AMPA ratio is considerably larger at PfN synapses than at cortical synapses onto ChIs, to start off with, in healthy mice. We were unable to discern a change in this ratio at cortical synapses after dopamine depletion, suggesting that this adaptation is selective to the thalamostriatal glutamatergic projection to ChIs. Strictly speaking, a reduction in an NMDA‐to‐AMPA ratio could mean either an increase in AMPA currents and/or a reduction in NMDA currents. Several facts lead us to prefer the latter interpretation. First, dopamine receptors are generally thought not to affect AMPAR trafficking in the striatum (Tritsch & Sabatini, 2012). Second, it was previously shown that whole‐cell NMDA currents triggered in ChIs with intrastriatal electrical stimulation (which should affect both cortical and thalamic glutamatergic fibers) were reduced in 6‐OHDA‐lesioned mice (Feng *et al*., 2014). Our findings complement that finding by demonstrating that this effect seems to be restricted to PfN synapses. Third, we found that D_5_R activation potentiates NMDA currents only at PfN synapses. This finding suggests that the absence of dopamine tone following 6‐OHDA treatment leads to an effective de‐potentiation of synaptic NMDA currents selectively for PfN afferents. Finally, our finding that the synaptic integration of PfN inputs is impaired after dopamine depletion is not consistent with an increase in AMPA currents.

### Comparison of PfN and cortical inputs to ChIs

*In vivo* striatal acetylcholine release from ChIs evoked by activation of glutamatergic PfN fibers is more strongly dependent on NMDARs than when evoked by activation of glutamatergic cortical fibers (Consolo *et al*., 1996a). Additionally, recent studies have shown that *in vitro* optogenetic activation of striatal glutamatergic fibers from either the PfN or the cortex can lead to dopamine release due to activation of nAChRs on dopamine fibers (Threlfell *et al*., 2012; Kosillo *et al*., 2016). Here too, the release of dopamine is more strongly dependent on NMDARs when triggered by activation of PfN fibers than when triggered by activation of cortical fibers (Kosillo *et al*., 2016). Our finding that the NMDA‐to‐AMPA ratio is higher at PfN synapses than at cortical synapses provides a direct (and first) demonstration of the neural substrate of this preferential dependency: Ionotropic glutamatergic PfN synapses onto ChIs have a larger NMDA component than their cortical counterparts.

We also compared these synapses with respect to other aspects of synaptic transmission. First, we replicated our previous findings, demonstrating that while cortical synapses onto ChIs are depressing—which usually indicates a synapse with a high presynaptic release probability—PfN synapses onto ChIs are facilitating—indicating that they are likely synapses with a lower release probability (Ding *et al*., 2010; Tanimura *et al*., 2016). Second, we compared the size of the unitary synaptic currents at these synapses and found that they were essentially equal. The most straightforward interpretation of this result is that individual synapses of each type express roughly the same amount of functional AMPARs. Thus, the well‐known asymmetry in the strength of these two glutamatergic pathways onto ChIs probably reflects a difference in the number and somatodendritic distribution of each of their respective synapses. PfN synapses are presumably more numerous and more proximally distributed than cortical synapses (Lapper & Bolam, 1992; Thomas *et al*., 2000; Ding *et al*., 2010).

A synapse, such as the PfN synapse onto ChIs, that is facilitating and has a large NMDA‐to‐AMPA ratio is optimized to integrate high‐frequency bursts of afferent input efficiently (Kosillo *et al*., 2016) (for brevity, we shall refer to synapses with these characteristics as *burst‐integrating* synapses). Thus, ChIs should be exceptionally tuned to bursts of input from the PfN. The PfN‐ChI projection is known to assign motivational value to unexpected, salient inputs (Matsumoto *et al*., 2001; Minamimoto & Kimura, 2002; Doig *et al*., 2014) in response to a sudden contextual change in the environment (Yamanaka *et al*., 2017). Thus, because the PfN responds to startling, salient input with a burst (Matsumoto *et al*., 2001), the burst‐integrating synapses onto ChIs are optimized to convey that burst to ChIs. ChIs, in turn, respond to thalamic input with a conditioned pause response, that assigns the motivational value (Kimura *et al*., 1984; Matsumoto *et al*., 2001; Goldberg & Reynolds, 2011; Doig *et al*., 2014), and thereby help in re‐directing attention to the changed context, and in changing the action selected (Matsumoto *et al*., 2001; Ding *et al*., 2010; Thorn & Graybiel, 2010; Aoki *et al*., 2015; Yamanaka *et al*., 2017).

Between context‐changing signals from the PfN, ChIs presumably receive cortical input conveying information regarding the ongoing processing of the cortico‐basal ganglia circuits. As autonomous pacemakers, ChIs are extremely sensitive to the precise timing of their afferent inputs (Bennett & Wilson, 1999). Depressing synapses that have a high probability of release are therefore optimized to form a high‐fidelity channel to convey the temporal structure of the cortical input, which is indeed more precisely timed than PfN input (Doig *et al*., 2014).

### Dopamine modulation of glutamatergic synapses in the striatum

ChIs express both D_5_ (D_1_‐like) and D_2_ receptors (Bergson *et al*., 1995; Yan & Surmeier, 1997). Previous studies have demonstrated that D_1_‐like receptors can potentiate (both synaptically localized and extrasynaptic) striatal NMDARs via a variety of mechanisms which involve phosphorylation, receptor trafficking, activation of protein kinases or the extracellular regulated kinase (ERK) pathway and even voltage‐activated calcium channels (Consolo *et al*., 1996b; Levine *et al*., 1996; Cepeda *et al*., 1998; Flores‐Hernandez, 2002; Hallett *et al*., 2005; Sarantis *et al*., 2009; Jocoy, 2011; Warre *et al*., 2011; Tritsch & Sabatini, 2012; Cahill *et al*., 2014). In line with these studies, we found that activation of D_5_Rs potentiates NMDA currents elicited by PfN stimulation in ChIs—but not by (nominally) cortical stimulation—providing a novel mechanism by which dopaminergic fibers can modulate PfN input. As mentioned above, we believe that this interaction is what leads to a reduction in NMDA currents after dopamine depletion.

Presynaptic dopamine D_2_ receptors can depress synaptic release probabilities at both glutamatergic projections to dorsal striatum (Nicola & Malenka, 1997; Hurd *et al*., 2001; Bamford *et al*., 2004; Rieck *et al*., 2004; Salgado *et al*., 2005; Yin & Lovinger, 2006; Higley & Sabatini, 2010; Tritsch & Sabatini, 2012) presumably also onto ChIs. While dopamine depletion failed to affect the probability of release in either of these glutamatergic projections onto ChIs in our acute slice preparation, further study is required—in preparations where dopamine tone is less compromised—to determine the putative influence of D_2_ receptors and dopamine depletion on release probabilities.

Previously, we found evidence for an increase in the release probability of PfN synapses on ChIs in a mouse model of HD, which may represent a homeostatic compensation for the loss of PfN fibers in HD (Tanimura *et al*., 2016). While there is also loss of PfN neurons in parkinsonism (Henderson *et al*., 2000; Halliday, 2009; Smith *et al*., 2014; Villalba *et al*., 2014), our data suggest that this loss does not drive a similar homeostatic response after dopamine depletion.

### Functional implications of the remodeling of PfN synapses after dopamine depletion

Ding *et al*. (2010) reported that when corticostriatal fibers are activated approximately 1 second after thalamostriatal fibers are, the temporal summation of cortical EPSPs in iSPNs is augmented. This augmentation was shown to require activation of postsynaptic M1 mAChRs on iSPNs, indicating that activation of ChIs by PfN is required to create this effect. This effect was not observed in dSPNs (Ding *et al*., 2010). Thus, PfN activation of ChIs creates a bias toward preferential activation of the indirect ‘No‐Go’ pathway. This conclusion is also supported by a recent study showing that activation of M4 mAChRs which are present only on dSPNs inhibits them, creating another mechanism by which activation of ChIs creates a bias toward preferential activation of the indirect pathway (Mamaligas & Ford, 2016), but see Hernandez‐Flores *et al*. (2014). Consequently, under normal conditions, ChIs seem to bias basal ganglia processing toward cessation of the current motor plan.

A recent study has shown that dopamine depletion leads to a selective weakening of the PfN projection to dSPNs, which will also bias the network toward a stronger relative activation of the indirect pathway (Parker *et al*., 2016). We in turn found that dopamine depletion leads to an effective weakening of the burst‐integrating character of PfN synapses onto ChIs by reducing the NMDA‐to‐AMPA ratio. We therefore propose that our finding represents a homeostatic response that counters the direct effect of dopamine depletion on the balance between the direct and indirect pathways and attempts to restore the balance by diminishing the burst‐integrating characteristics of PfN synapses.

Weakening of the burst‐integrating character of the PfN synapse onto ChIs could also help explain the diminution of the conditioned pause response of the tonically active neurons of the striatum [which correspond primarily to ChIs (Aosaki *et al*., 1995)] in dopamine‐depleted primates (Kimura *et al*., 1984; Aosaki *et al*., 1994). Because the conditioned response requires an intact parafascicular projection to striatum, it is possible that the weakening of this pathway following dopamine depletion that we describe herein will also tend to diminish the conditioned pause response. Finally, because this projection promotes the changing of motor plans (Ding *et al*., 2010; Thorn & Graybiel, 2010; Aoki *et al*., 2015; Yamanaka *et al*., 2017), it is possible that the weakening of this projection contributes to the akinetic nature of Parkinson's disease.

## Conflict of Interest statement

The authors declare no conflict of interest.

## Author contribution

JJAB conducted the experiments, analyzed the data, prepared the figures and prepared the manuscript. LT wrote code for data analysis. W‐HC conducted some experiments. JAG designed the experiments, oversaw the work, prepared the figures and wrote the manuscript.

**Abbreviations**
6‐OHDA: 6‐hydroxydopamine
AAVs: adeno‐associated virus(es)
ACSF: artificial cerebrospinal fluid
AMPAR: α‐Amino‐3‐hydroxy‐5‐methyl‐4‐isoxazolepropionic acid receptor
ancova: analysis of covariance
ChI: cholinergic interneuron
ChR2: channelrhodopsin‐2
d/iSPN: direct/indirect pathway spiny neurons
D‐APV: D‐(‐)‐2‐Amino‐5‐phosphonopentanoic acid
EPSC/P: excitatory postsynaptic current/potential
eYFP: enhanced yellow fluorescent protein
GABA: γ‐Aminobutyric acid
GC: genome copies
HD: Huntington's disease
LED: light‐emitting diode
m/nAChR: muscarinic/nicotinic acetylcholine receptor
NMDAR: N‐Methyl‐D‐aspartic acid receptor
PD: Parkinson's disease
PfN: parafascicular nucleus
PPR: paired‐pulse ratio
RST: two‐tailed Wilcoxon rank‐sum test for independent samples
SRT: two‐tailed Wilcoxon signed‐rank test for paired samples
TEA: tetraethylammonium
TH: tyrosine hydroxylase

## Data Availability

All data files and code to extract and analyze the data will be provided freely and promptly upon receipt of a written request.
