# Supplementary material for: Selective remodeling of glutamatergic transmission to striatal cholinergic interneurons after dopamine depletion
Source: Eur J Neurosci. 2017 Oct 6;49(6):824–33. doi: 10.1111/ejn.13715 (PMC6519226; doi:10.1111/ejn.13715)
Supplement: Supplementary file 1 [file EJN-49-824-s001.pdf]

EJN European Journal of Neuroscience FENS Federation of European Neuroscience Societies

Selective remodeling of glutamatergic transmission to striatal cholinergic interneurons after dopamine depletion

Jose de Jesus Aceves Buendia, Lior Tiroshi, Wei-Hua Chiu & Joshua A. Goldberg

Review timeline:

Submission date:	12 July 2017
Editorial Decision:	04 August 2017
Revision received:	22 August 2017
Editorial Decision:	11 September 2017
Revision received:	13 September 2017
Accepted:	13 September 2017

Editor: Paul Bolam

1st Editorial Decision 04 August 2017

Dear Josh,

Your manuscript has been reviewed by three external reviewers as well as by the Editorial team.

As you can see, the reviewers like the study and are keen to see it published in EJN. However, each of them raises a few points that need to be addressed. These mostly will simply require clarification of the text and constitute 'minor revisions'. A couple of points stand out, particularly the issue of the quantification of the lesions and the reporting of the cortical data. Please address these very carefully as well as the other points that they raise. We also noted the following points that should also be addressed.
- Dosage of ketamine–xylazine for terminal anaesthesia?
- Dilution of secondary antibody for histology?
- Please ensure that the reporting of statistical data adheres to EJN guidelines, notable please report the precise values of P.
- Please carefully proof-read the references, some are not quite in EJN style.

If you are able to respond fully to the points raised, we would be pleased to receive a revision of your paper within 30 days.

Thank you for submitting your work to EJN and support of this Special Issue associated with IBAGS.

Kind regards,

Paul & John
co-Editors in Chief, EJN

Reviews:

Reviewer: 1 (Paul Apicella, Aix Marseille Université, France)

Comments to the Author
The influence of thalamic afferents on the striatal circuity is a hot topic in basal ganglia pathophysiology. There is a strong emphasis on the relationship between the glutamatergic projection from the intralaminar nuclei of the thalamus (i.e., the rodent parafascicular nucleus or PfN) to the cholinergic interneurons (ChIs) that play an important role in regulating striatal output. In this study, Aceves Buendia et al. examined the impact of glutamatergic thalamic projections to striatal ChIs in mice with experimentally induced dopamine (DA) depletion. To do this, they combined optogenetics and patch-clamp recordings in acute striatal slices from control and DA-depleted transgenic mice. For comparison, they also analyzed the effects of the DA depletion on glutamatergic cortical projections to striatal ChIs. This analysis revealed changes in synaptic transmission at PfN synapses after 6-OHDA treatment, with a reduction in the NMDA/AMPA ratio reflecting an impaired synaptic integration of PfN inputs to ChIs. The authors found other evidence to show that DA

depletion leads to downregulation of NMDA currents in DA-depleted mice. Interestingly, there is no change in the NMDA/AMPA ratio at cortical synapses after DA depletion, suggesting that this adaptation is selective to the thalamostriatal pathway linking the PfN with ChIs.
Overall, the contribution of this in vitro work is to further demonstrate that thalamic modulation of ChIs could support the important role of the thalamostriatal pathway for learning and behavioral flexibility which has been evidenced by studies in behaving animals. This is a report about which, in my opinion, there is very little to critique. The data as a whole are interesting and clearly presented, and the explanation advanced for the reported results is rather satisfactory.

Major comments
Point 1.
The authors used unilateral injections of 6-OHDA in the MFB to induce DA depletion in the striatum. I am a little bothered by the fact that the extent of DA depletion was not very detailed. It is said in the Materials and Methods section that TH immunoreactivity has been analyzed on striatal slices, but the authors did not provide quantitative results. There was only one picture illustrating the DA depletion (Fig.1C).

Point 2.
I don't have the expertise to assess appropriateness of patch-clamp methodology. This needs to be check by an expert in the field.

Minor comments
Typo. Intro, p.3: ... a functional re-wiring of thalmostriatal projection to SPNs in PD

Reference. Results, p.10: Previous work has shown that intrastriatal acetylcholine (Consolo, Baldi, et al. should be Consolo et al.)

Reviewer: 2 (James Tepper, Rutgers University, USA)

Comments to the Author
The ms. by Aceves Bueneda and colleagues describes the effects of 6-OHDA lesioning of the nigrostriatal pathway on the kinetics of glutamatergic EPSCs in striatal cholinergic interneurons evoked by optogenetic stimulation of fibers arising from cortex or the thalamic parafascicular nucleus in two different strains of transgenic mice. The main results are that dopamine depletion failed to affect short term plasticity or quantal properties of either corticostriatal or thalamostriatal EPSCs. However, there was a significant difference in the NMDA/AMPA ratio of the corticostriatal and thalamostriatal EPSCs with thalamic inputs exhibiting a significantly larger NMDA/AMPA ratio. The NMDA/AMPA ratio was reduced by 6-OHDA to thalamic but not cortical inputs, and application of a D5 agonist increased the NMDA component of the thalamic input to cholinergic interneurons (cortical inputs were not tested). The authors conclude that a reduction in the NMDA component of the thalamostriatal input to cholinergic interneurons represents a homeostatic compensation for the effects of dopamine depletion on the balance between the direct and indirect pathways.

The ms. is concise and well written. The literature reviews in the introduction and discussion are balanced and fair. The recordings look good and the statistical analyses are appropriate. The relevance for the aims and scope of EJN and compliance etc. seem fine. I have only a few comments/criticisms.

1. There is a relative paucity of experimental data with respect to the 6-OHDA effect on the cortical input compared to an overemphasis on data arising from thalamic stimulation. In several places, the ms, simply says "data not shown" where 6-OHDA lesions are shown in figures and analyses to affect the thalamic input but no effect on the cortical input. It is not as if the paper is overloaded with data (there are only 5 relatively simple figures). All of these data ought to be shown just as they are for the thalamic stimulation.

2. Similarly, the authors examine the effects of a D5 agonist on the thalamic evoked NMDA current but not on the cortically evoked NMDA current, and then do some create a weak argument in the discussion. Why is this? It is simple enough and absolutely necessary to test the effects of SKF-81297 on NMDA currents elicited by both cortical and thalamic inputs.

3. 6-OHDA lesions shorten the decay time of the thalamic EPSC but are alleged (this is another example of the "data not shown") to have no effect in the kinetics of the cortically evoked EPSC. This is followed (p.9) by a discussion of AMPA subunit composition implying the that reduction in decay time is due to a change in AMPA receptors. But 6-OHDAreduces the NMDA/AMPA ratio of this response. Why couldn't a reduction in NMDA current be responsible for the change in decay time?

Reviewer: 3 (Stephanie Cragg, University of Oxford, UK)

Comments to the Author
This manuscript by Aceves et al is a well-written study of good quality and well within the scope of the journal. It investigates the impact of a 6-OHDA-inducd depletion of dopamine on NMDA and AMPA inputs to striatal cholinergic interneurons, with a view to understanding changes to interneuron excitability in Parkinson's disease. The authors use optogenetics in mice to activate either cortical or thalamic inputs and they corroborate previous recent findings that NMDA and AMPA currents differ between cortical and thalamic inputs. They show further that NMDA:AMPA current properties change after dopamine is depleted: EPSC decay time is reduced, and the NMDA:AMPA current ratio becomes reduced for thalamic but not cortical inputs. This change could be restored by applied by a D1/D5 agonist SKF-81297. Drawing also from other findings in the literature, the authors speculate that the reduced NMDA:AMPA ratio is likely due to a reduction of the NMDA component rather than a change to the AMPA current, and that it will impact on striatal integration in PD. This study is nicely put together and well conducted.

Issues for the authors to address:
1. The authors use voltage clamp to quantify NMDA: AMPA currents in cholinergic interneurons. Holding cells at -70 and +40 mV with Cs internal solution is a standard measurement of AMPA and NMDA currents, but cholinergic interneurons, which have large soma and vast and remote dendritic arbours, are unlikely to be well clamped under these conditions (Williams & Mitchell, 2014, Nat Neurosci), which weakens the accuracy of the quantifications made here. However, as the authors performed all experiments under the same conditions, the comparison of NMDA: AMPA ratio is nonetheless meaningful. The authors should rather mention this caveat of their recordings within the manuscript.

2. Figure 1, Can the authors add an image for ChR2 expression in cortical inputs as well as thalamic?

3. P8-9, and also Discussion. The authors "found no evidence that dopamine depletion alters the presynaptic probability of release at either glutamatergic synapse onto ChIs". However, all of these recordings were conducted in ex vivo conditions, in brain slices, when tonic dopamine release is minimal, and would not be expected to lead to significant activation of D2 receptors at either the first or second pulse within the short interval used here. The inter-pulse interval appears to be 100 ms (not stated anywhere?) and for D2 regulation of PPR for release of dopamine at least, this is too short an interval to see much D2 influence (see Phillips-PEM 2002 Synapse; Schmitz-Y et al 2002, J Neurosci; Cragg-SJ 2003 J Neurosci). PPR intervals of at least 200 ms, and ideally 500 ms are needed to expose an effect of dopamine on PPR for dopamine release, and would presumably also be needed to see effects of dopamine depletion. Please could the authors rework their description and interpretations since their findings cannot be used to indicate a role for D2 receptors in the regulation of glutamatergic inputs.

4. It would be useful if the cortex data summarized in figure 4 were illustrated as for thalamus (especially as the authors have already acquired the data).

5. The discussion is a little long and could be more concise

Minor comments
6. Terminology: The authors introduce an abbreviation for the NMDA:AMPA ratio - N2Ar. This acronym reads like a chemical structure and hinders the reading of the manuscript and is not necessary. The field normally readily use terms such as NMDA:AMPA ratio (or AMPA/NMDA ratio) without introducing an acronym. Please can we suggest that the authors delete this acronym.

7. Abstract. "ChI's D5 receptors". Better to rephrase as "D5 receptors on ChIs".

8. The terms "optical paired-pulse ratios", and "optical ratios" are misleading. The data to which they refer are not optical ratios but current ratios. Better to say evoked paired-pulse ratios or similar.

9. Kosillo et al. used 10 pulses, not 12 pulses as cited, at 25 Hz.

10. P8 Methods: Since different studies using 6-OHDA injections use a range of different types of control that include sham/vehicle-injected animals/hemispheres or simply non-injected hemispheres, could the authors please clarify explicitly and as appropriate in the methods that their control mice are those injected with AAV but not given an MFB injection of 6-OHDA.

11. P8. Please specify inter-pulse intervals used for paired-pulse studies. This information does not appear to be stated anywhere within text or legends.

12. P9. Section Title. "Synaptic decay times" is not very accurate. Please rephrase e.g. "EPSC decay times"

13. Discussion “Comparison of PfN and cortical inputs to ChIs”. The authors discuss that the facilitating PfN-ChI inputs might be better tuned to bursts of input, whereas the depressing synapses of the cortical input mght be better tuned to be high fidelity to convey better temporal information. This point was also made previously by Kosillo et al 2016, which it would be appropriate to cite in this context, and which also showed a different latency and duration of activation of ChIs by these different inputs.

14. Could authors please label the holding voltage in figure 2, 3, and 4.

15. P12. It would be useful if authors could clarify that SKF-81297 acts on D1/D5 receptors in first paragraph.

Authors’ Response 22 August 2017

Editors’ comments:

Comment:
As you can see, the reviewers like the study and are keen to see it published in EJN. However, each of them raises a few points that need to be addressed. These mostly will simply require clarification of the text and constitute 'minor revisions'. A couple of points stand out, particularly the issue of the quantification of the lesions and the reporting of the cortical data. Please address these very carefully as well as the other points that they raise.

Response:
In the revised manuscript, we have quantified the extent of reduction in tyrosine hydroxylase (TH) immunoreactivity in the 6-OHDA lesioned striata (we have also added to Figure 1 new images of ChR2-laden terminals and TH immunoreactivity from the transgenic mice expressing ChR2 under the Thy1-promoter, as requested by the reviewers).
As requested, we have added new data concerning the cortical projection to cholinergic interneurons (ChIs), which include: a) NMDA-to-AMPA ratios before and after the 6-OHDA lesion; and b) the effect (or, rather, the lack thereof of an effect) of the D1-like agonist on NMDA currents. These data now complete the comparison of synaptic transmission at PfN and cortical projection in terms of paired-pulse ratios (PPRs), minimal stimulation, and NMDA-to-AMPA ratios. The revised manuscript thus strengthens the conclusion regarding the distinction between these two afferent glutamatergic synapses, and the selective adaptation of NMDA currents that occurs solely in parafascicular nucleus (PfN) projections to ChIs. Consequently, we have revised the title of the manuscript to better represent this more complete exposition.
The adaptation of the NMDA currents at PfN synapses following 6-OHDA warranted the quantification of its impact on synaptic integration which was done for two stimulation frequencies (10 and 25 Hz) and with some pharmacology, and is represented in Figure 5 (and the accompanying text). Because we found no adaptation in the NMDA currents in cortical synapses after 6-OHDA lesions this “second-order” characterization of synaptic integration at these synapses was not warranted, and we do not have these data.

Comment:
We also noted the following points that should also be addressed.
- Dosage of ketamine–xylazine for terminal anaesthesia?
- Dilution of secondary antibody for histology?
- Please ensure that the reporting of statistical data adheres to EJN guidelines, notable please report the precise values of P.
- Please carefully proof-read the references, some are not quite in EJN style.

Response:
Done. Note that for Wilcoxon tests the P-values are often rational numbers. Because EJN is asking for the precise value, we’ve provided the precise ratio when possible.

Reviewer 1 comments:

Comment:
The influence of thalamic afferents on the striatal circuity is a hot topic in basal ganglia pathophysiology. There is a strong emphasis on the relationship between the glutamatergic projection from the intralaminar nuclei of the thalamus (i.e., the rodent parafascicular nucleus or PfN) to the cholinergic interneurons (ChIs) that play an important role in regulating striatal output. In this study, Aceves Buendia et al. examined the impact of glutamatergic thalamic projections to striatal ChIs in mice with experimentally induced dopamine (DA) depletion. To do this, they combined optogenetics and patch-clamp recordings in acute striatal slices from control and DA-depleted transgenic mice. For comparison, they also analyzed the effects of the DA depletion on glutamatergic cortical projections to striatal ChIs. This analysis revealed changes in synaptic transmission at PfN synapses after 6-OHDA treatment, with a reduction in the NMDA/AMPA ratio reflecting an impaired synaptic integration of PfN inputs to ChIs. The authors found other evidence to show that DA depletion leads to downregulation of NMDA currents in DA-depleted mice. Interestingly, there is no change in the NMDA/AMPA ratio at cortical synapses after DA depletion, suggesting that this adaptation is selective to the thalamostriatal pathway linking the PfN with ChIs.
Overall, the contribution of this in vitro work is to further demonstrate that thalamic modulation of ChIs could support the important role of the thalamostriatal pathway for learning and behavioral flexibility which has been evidenced by studies in behaving animals. This is a report about which, in my opinion, there is very little to critique. The data as a whole are interesting and clearly presented, and the explanation advanced for the reported results is rather satisfactory.

Major comments
Point 1.
The authors used unilateral injections of 6-OHDA in the MFB to induce DA depletion in the striatum. I am a little bothered by the fact that the extent of DA depletion was not very detailed. It is said in the Materials and Methods section that TH immunoreactivity has been analyzed on striatal slices, but the authors did not provide quantitative results. There was only one picture illustrating the DA depletion (Fig.1C).

Response:
Thank you for the overall positive appraisal of our manuscript. We have now added a quantification of the degree of the lesion, which is explained in the Methods section and described in the Results section. Because the difference in intensity of immunofluorescence between the lesioned and control striata was so immense we were unable to generate images in which both intensities fell within the dynamic range of the image (even after taking 16 bit images). We therefore had to revert to quantifying the number of pixels (out of roughly 200,000) that were excessively bright within a set area of striatum (in comparison to background intensity, estimated from a region of the slice that is nominally devoid of TH immunoreactivity). The difference was so incredibly large (96% bright pixels in control slices vs. 0.015% pixels in the lesion slices, i.e. 4 orders of magnitude in the number of pixels) that we report them in the text only (because any visual rendition of the result looks silly).

Comment:
Point 2.
I don't have the expertise to assess appropriateness of patch-clamp methodology. This needs to be check by an expert in the field.

Minor comments
Typo. Intro, p.3: ... a functional re-wiring of thalmostriatal projection to SPNs in PD

Reference. Results, p.10: Previous work has shown that intrastriatal acetylcholine (Consolo, Baldi, et al. should be Consolo et al.)

Response:
Corrected.

Reviewer 2 comments:

Comment:

The ms. by Aceves Bueneda and colleagues describes the effects of 6-OHDA lesioning of the nigrostriatal pathway on the kinetics of glutamatergic EPSCs in striatal cholinergic interneurons evoked by optogenetic stimulation of fibers arising from cortex or the thalamic parafascicular nucleus in two different strains of transgenic mice. The main results are that dopamine depletion failed to affect short term plasticity or quantal properties of either corticostriatal or thalamostriatal EPSCs. However, there was a significant difference in the NMDA/AMPA ratio of the corticostriatal and thalamostriatal EPSCs with thalamic inputs exhibiting a significantly larger NMDA/AMPA ratio. The NMDA/AMPA ratio was reduced by 6-OHDA to thalamic but not cortical inputs, and application of a D5 agonist increased the NMDA component of the thalamic input to cholinergic interneurons (cortical inputs were not tested). The authors conclude that a reduction in the NMDA component of the thalamostriatal input to cholinergic interneurons represents a homeostatic compensation for the effects of dopamine depletion on the balance between the direct and indirect pathways.

The ms. is concise and well written. The literature reviews in the introduction and discussion are balanced and fair. The recordings look good and the statistical analyses are appropriate. The relevance for the aims and scope of EJN and compliance etc. seem fine. I have only a few comments/criticisms.

1. There is a relative paucity of experimental data with respect to the 6-OHDA effect on the cortical input compared to an overemphasis on data arising from thalamic stimulation. In several places, the ms, simply says "data not shown" where 6-OHDA lesions are shown in figures and analyses to affect the thalamic input but no effect on the cortical input. It is not as if the paper is overloaded with data (there are only 5 relatively simple figures). All of these data ought to be shown just as they are for the thalamic stimulation.

Response:
Thank you for the overall positive assessment of the manuscript. As stated above in the response to the editors, we have added the missing data concerning the cortical projection to ChIs, so that there is now a complete set of data concerning the three quantities we measured to characterized synaptic transmission: PPRs, minimal stimulation and NMDA-to-AMPA ratios. The manuscript now has only one legitimate "not shown" statement regarding the comparison of the amplitude of the EPSCs among various stereotaxically injected mice is not valid due to the variability in transfection.

Comment:
2. Similarly, the authors examine the effects of a D5 agonist on the thalamic evoked NMDA current but not on the cortically evoked NMDA current, and then do some create a weak argument in the discussion. Why is this? It is simple enough and absolutely necessary to test the effects of SKF-81297 on NMDA currents elicited by both cortical and thalamic inputs.

Response:
We are truly grateful for this criticism, as it really improved the manuscript. You are right: this was an easy and straightforward experiment which we have now conducted. Interestingly, we found that NMDA currents are not potentiated at cortical synapses (while they are at PfN synapses). Thus, this demonstrate that D5 receptors are coupled to NMDARs selectively at PfN synapses which is a novel and interesting finding, and could perhaps shed a new light on the effect of dopamine depletion on thalamostriatal projection to ChIs.

Comment:
3. 6-OHDA lesions shorten the decay time of the thalamic EPSC but are alleged (this is another example of the "data not shown") to have no effect in the kinetics of the cortically evoked EPSC. This is followed (p.9) by a discussion of AMPA subunit composition implying the that reduction in decay time is due to a change in AMPA receptors. But 6-OHDAreduces the NMDA/AMPA ratio of this response. Why couldn't a reduction in NMDA current be responsible for the change in decay time?

Response:
This too was an excellent criticism. A closer look at the data suggest that not only is this interpretation possible, we now think it is likely the correct one. As a consequence, and because we present more direct and stronger evidence for the reduction in NMDA current after 6-OHDA, we have decided to entirely withdraw the claims regarding changes in the EPSC kinetics from the manuscript.

Reviewer 3 comments:

Comment:
This manuscript by Aceves et al is a well-written study of good quality and well within the scope of the journal. It investigates the impact of a 6-OHDA-inducd depletion of dopamine on NMDA and AMPA inputs to striatal cholinergic interneurons, with a view to understanding changes to interneuron excitability in Parkinson's disease. The authors use optogenetics in mice to activate either cortical or thalamic inputs and they corroborate previous recent findings that NMDA and AMPA currents differ between cortical and thalamic inputs. They show further that NMDA:AMPA current properties change after dopamine is depleted: EPSC decay time is reduced, and the NMDA:AMPA current ratio becomes reduced for thalamic but not cortical inputs. This change could be restored by applied by a D1/D5 agonist SKF-81297. Drawing also from other findings in the literature, the authors speculate that the reduced NMDA:AMPA ratio is likely due to a reduction of the NMDA component rather than a change to the AMPA current, and that it will impact on striatal integration in PD. This study is nicely put together and well conducted.
Issues for the authors to address:
1. The authors use voltage clamp to quantify NMDA: AMPA currents in cholinergic interneurons. Holding cells at -70 and +40 mV with Cs internal solution is a standard measurement of AMPA and NMDA currents, but cholinergic interneurons, which have large soma and vast and remote dendritic arbours, are unlikely to be well clamped under these conditions (Williams & Mitchell, 2014, Nat Neurosci), which weakens the accuracy of the quantifications made here. However, as the authors performed all experiments under the same conditions, the comparison of NMDA: AMPA ratio is nonetheless meaningful. The authors should rather mention this caveat of their recordings within the manuscript.

Response:
Thank you for being supportive of the manuscript. While we obviously concur that the space clamp is a tricky business, this cesium-loaded voltage clamp assay is a standard one, which is used ubiquitously on other dendritic neurons in slices, and we and others (Ben Bennett and Charlie Wilson) have used it previously to publish measurements of EPSCs in ChIs. Consequently, we feel that it would be wrong to belabor this point, particulary because, as you have astutely pointed out, we are comparing two conditions using the same assay so that it has no impact on our finding.

Comment:
2. Figure 1, Can the authors add an image for ChR2 expression in cortical inputs as well as thalamic?

Response:
Done.

Comment:
3. P8-9, and also Discussion. The authors "found no evidence that dopamine depletion alters the presynaptic probability of release at either glutamatergic synapse onto ChIs". However, all of these recordings were conducted in ex vivo conditions, in brain slices, when tonic dopamine release is minimal, and would not be expected to lead to significant activation of D2 receptors at either the first or second pulse within the short interval used here. The inter-pulse interval appears to be 100 ms (not stated anywhere?) and for D2 regulation of PPR for release of dopamine at least, this is too short an interval to see much D2 influence (see Phillips-PEM 2002 Synapse; Schmitz-Y et al 2002, J Neurosci; Cragg-SJ 2003 J Neurosci). PPR intervals of at least 200 ms, and ideally 500 ms are needed to expose an effect of dopamine on PPR for dopamine release, and would presumably also be needed to see effects of dopamine depletion. Please could the authors rework their description and interpretations since their findings cannot be used to indicate a role for D2 receptors in the regulation of glutamatergic inputs.

Response:
Thank you for this criticism. We removed the sentence from the Results section and tempered the Discussion. It now reads: "Presynaptic dopamine D_2 receptors can depress synaptic release probabilities at both glutamatergic projections to dorsal striatum (Nicola & Malenka, 1997; Hurd *et al.*, 2001; Bamford *et al.*, 2004; Rieck *et al.*, 2004; Salgado *et al.*, 2005; Yin & Lovinger, 2006; Higley & Sabatini, 2010; Tritsch & Sabatini, 2012) presumably also onto ChIs. While dopamine depletion failed to affect the probability of release in either of these glutamatergic projections onto ChIs in our acute slice preparation, further study is required – in preparations where

dopamine tone is less compromised – to determine the putative influence of D_2 receptors and dopamine depletion on release probabilities."

Comment:
4. It would be useful if the cortex data summarized in figure 4 were illustrated as for thalamus (especially as the authors have already acquired the data).

Response:
As explained in the response to the editors we added the data concerning NMDA-to-AMPA ratios at cortical synapses after 6-OHDA treatment (and also added new data concerning the lack of any effect of the D1-like receptor agonist on NMDA currents in these synapses, as requested by reviewer 3). The manuscript now presents a more complete comparison of synaptic transmission between PfN and cortical synapses onto ChIs. Because there was no observed change in the NMDA-to-AMPA ratio at cortical synapses, we felt that measuring the effect of 6-OHDA on synaptic integration (in current clamp) in the Thy1-ChR2 mice was not warranted. Thus, these experiments were not carried out, because we expect a negative result. We feel our data are timely now as conducted, without the significant delay collecting these negative results would incur.

Comment:
5. The discussion is a little long and could be more concise

Response:
The Discussion has been shortened by 200 words.

Comment:
Minor comments
6. Terminology: The authors introduce an abbreviation for the NMDA:AMPA ratio - N2Ar. This acronym reads like a chemical structure and hinders the reading of the manuscript and is not necessary. The field normally readily use terms such as NMDA:AMPA ratio (or AMPA/NMDA ratio) without introducing an acronym. Please can we suggest that the authors delete this acronym.

Response:
Done.

Comment:
7. Abstract. "ChI's D5 receptors". Better to rephrase as "D5 receptors on ChIs".

Response:
Corrected.

Comment:
8. The terms "optical paired-pulse ratios", and "optical ratios" are misleading. The data to which they refer are not optical ratios but current ratios. Better to say evoked paired-pulse ratios or similar.

Response:
We feel it is important to emphasize *how* the stimulation is delivered and therefore changed the terminology to "optogenetic paired-pulse ratios".

Comment:
9. Kosillo et al. used 10 pulses, not 12 pulses as cited, at 25 Hz.

Response:
This is a fine point. We feel that it is more important to give those authors the credit of using to stimulation frequencies which we emulated in this study.

Comment:
10. P8 Methods: Since different studies using 6-OHDA injections use a range of different types of control that include sham/vehicle-injected animals/hemispheres or simply non-injected hemispheres, could the

authors please clarify explicitly and as appropriate in the methods that their control mice are those injected with AAV but not given an MFB injection of 6-OHDA.

Response:

Thank you for this comment. We have now clarified what control we used. The extra sentence in the Methods section reads: "Non-lesioned mice served as controls."

Comment:

11. P8. Please specify inter-pulse intervals used for paired-pulse studies. This information does not appear to be stated anywhere within text or legends.

Response:

This was already mentioned in the Methods section of the original submission under the subtitle: "Slice visualization, electrophysiology and optogenetic stimulation".

Comment:

12. P9. Section Title. "Synaptic decay times" is not very accurate. Please rephrase e.g. "EPSC decay times"

Response:

This topic was pulled from the manuscript.

Comment:

13. Discussion "Comparison of PfN and cortical inputs to ChIs". The authors discuss that the facilitating PfN-ChI inputs might be better tuned to bursts of input, whereas the depressing synapses of the cortical input might be better tuned to be high fidelity to convey better temporal information. This point was also made previously by Kosillo et al 2016, which it would be appropriate to cite in this context, and which also showed a different latency and duration of activation of ChIs by these different inputs.

Response:

We have added an appropriate reference to Kosillo et al. 2016.

Comment:

14. Could authors please label the holding voltage in figure 2, 3, and 4.
15. P12. It would be useful if authors could clarify that SKF-81297 acts on D1/D5 receptors in first paragraph.

Response:

Done.

2nd Editorial Decision 11 September 2017

Dear Josh,

Thank you for the revised version of your paper. It has now been seen by two of the original the reviewers and we are pleased to say that it will be accepted for publication in EJN after you have dealt with a couple of points. Please supply a 'clean' version of the manuscript and correct the spelling as indicated by the reviewer.

If you are able to respond fully to the points raised, we shall be pleased to receive a revision of your paper within 30 days.

Thank you for submitting your work to EJN and support of this Special Issue.

Kind regards,

Paul and John
co-Editors in Chief, EJN

Reviews:

EJN European Journal of Neuroscience FENS Federation of European Neuroscience Societies

Reviewer: 2 (James Tepper, Rutgers University, USA)

Comments to the Author
The authors' responses to my criticisms and comments are appropriate and sufficient.
I have nothing else to add other than "Parafasicluar" (sic) is misspelled in the graphical abstract.

Reviewer: 1 (Paul Apicella, Aix Marseille Université, France)

Comments to the Author
The authors have addressed adequately my concerns about quantification of the 6-0HDA-induced lesion. I am fully satisfied with the revised version and have no further comments on the manuscript.

Authors' Response 13 September 2017

Comment:

Reviewer: 2

Comments to the Author
The authors' responses to my criticisms and comments are appropriate and sufficient.
I have nothing else to add other than "Parafasicluar" (sic) is misspelled in the graphical abstract.

Reviewer: 1

Comments to the Author
The authors have addressed adequately my concerns about quantification of the 6-0HDA-induced lesion. I am fully satisfied with the revised version and have no further comments on the manuscript.

Response:
Corrected. Thank you!
